# Editorial: Lessons from COVID-19: Building a fairer, healthier, inclusive and sustainable post-pandemic society

**DOI:** 10.3389/fsoc.2022.1100563

**Published:** 2022-12-06

**Authors:** Kath Woodward, Hannah Bradby, Guillermina Jasso, Sin Yi Cheung

**Affiliations:** ^1^The Open University, Milton Keynes, United Kingdom; ^2^Uppsala University, Uppsala, Sweden; ^3^New York University, New York, NY, United States; ^4^School of Social Sciences, Cardiff University, Cardiff, United Kingdom

**Keywords:** COVID-19, pandemic, social structures, inequalities, politics

During 2020 and 2021, there was a surge of research and commentary produced in response to the spread of the novel coronavirus COVID-19, and the associated public health responses. Significant research effort went into charting the progress of the infections, as well as developing therapeutic and preventative measures against those infections. As the early research tried to make sense of the immediate effects of the virus on the body and on society, now we are in a position to take a somewhat more considered view of the effects of the pandemic. These reflections include contributions from the social sciences about the socioeconomic and sociocultural contexts in which the pandemic was experienced and managed and the social factors which influenced behavior and reaction to state policies. Crucially, we can also begin to consider the lessons that we can learn from our experience of and responses to the COVID-19 virus, and how these can inform our progressive reform of societies and notably the inequalities which were highlighted during the pandemic. The papers that came in for this Research Topic reflect sociological preoccupations with how social structures have influenced the effects of the pandemic in ways that are both protective and injurious. In this collection, the papers include explorations of how social ties and social capital, equity, gender and racism have played out in the pandemic to date. With a particular focus on the UK, two papers consider governance and the political implications of social order.

Below we index these papers briefly, starting with Snel et al. who offer an examination of how social capital protected against the well documented negative impacts on mental health during the pandemic in the Netherlands. Association and multiple step linear regression for a weighted panel survey of 22,696 adults showed that the negative impact on mental health increased over the year 2020. Women, young people and those with low income and poor self-assessed health experienced more fear and more stress, but this was mediated by social capital, defined as trust in institutions and other people as well as participating in solidarity networks.

Looking at the effect of the pandemic on people's social ties, Bertogg and Koos analyse a quota sample of 3,378 individuals, weighted to ensure representation of the whole German population, using multinomial logistic regression, to show both loss and gain of strong and of weak ties. The lockdown period required restriction of contact and was associated with a shrinkage of social network for 1 in 3 people, in terms of losing touch with a friend of acquaintance. However, 1 in 6 reported gaining social ties. The volatility of networks was linked to people's gender, migration background and level of education.

Turning to issues of equity and justice, Karlsen and Nelson use a survey responses and interviews to consider the lived experience of minoritized people in England during the first pandemic lockdown. Frustration with a corrupt and incompetent public response to COVID-19, combined with concerns that racism was intensifying the risks to ethnic minorities, served to undermine confidence in and preparedness to participate in local community involvement. Recognition of the particularly damaging effects of the pandemic-experience of minoritized groups is a crucial step toward rebuilding trust, which in turn is necessary before identifying transformative policy change for a better post-pandemic society for all.

Equity on a global scale preoccupies (Ahlberg and Bradby's) consideration of global inequalities in access to COVID-19 vaccinations. Rich countries' failure to make vaccinations available to poor countries is examined as an effect of economic and political structures of financialized capitalism which re-inscribe disparities in access to basic medications, including the COVID-19 vaccination. Neoliberal deregulation, along with the corporate financialization aspects of the pharmaceutical and medical devices industry, are aspects of healthcare provision that sociologists need to interrogate in order to understand how the pandemic has compounded inequity.

The gendered differentiation of the effects of the pandemic is picked up in three papers. O'Sullivan et al. offer evidence that the negative effects of the pandemic public health restrictions were marked among women, and particularly mothers, to a greater extent than fathers. Drawing on an online survey of 346 parents, they show that mothers reported themselves ten times more likely to be responsible for home schooling during the lockdown in Ireland, and link this to mothers' greater likelihood of reporting themselves to be more stressed than the fathers (*n* = 132) completing the survey.

de Sousa et al. explore how the conditions of the pandemic exaggerated hegemonic male behaviors in a way that was health damaging. They analyze 50 men's responses to a semi-structured online survey with a collective subject discourse method, demonstrating the inequitable balance of power along gendered axes, which were largely disadvantageous to women.

Hamzah et al.s' online survey exploring how self-assessed career success was related to self-efficacy and perception of organizational support during the lockdown in Malaysia. The survey data from 146 women managers were analyzed using multiple linear regressions to show that a perception of supportive management was positively associated with subjective career success.

With a particular focus on the UK, Scambler describes the devastating effects of the COVID-19 pandemic, hard on the heels of a decade of austerity politics. He calls attention to the classic sociological issues of social order and change, as a means to rediscover the discipline's mission to promote the good society, a target which cannot be achieved by further disadvantaging the poor and vulnerable.

May reviews the relationship between science and politics during the response to the spread of COVID-19, focused on England. In tracing tensions between science and politics, May identifies a spectator democracy, characterized by passive citizens and calls for a development of civic epistemologies as an appropriate reform to the atomization of society that the pandemic accelerated.

And finally, Outhwaite reviews *Pandemics, Politics and Society: Critical Perspectives*, edited by Gerard Delanty that makes an early assessment of the social transformation that the COVID-19 pandemic represents, putting COVID-19 in the context of the history of pandemics and providing a broad analysis.

These wide ranging articles deploying a range of different methodological and theoretical frameworks demonstrate the importance of critical social science in making sense of a global event which unsurprisingly sought scientific solutions, for example in the form of vaccines, but which also has extensive social, economic and political implications.

## Author contributions

All authors listed have made a substantial, direct, and intellectual contribution to the work and approved it for publication.

